# Predicting competitive outcomes in professional e-sports from 60-second pre-match voice acoustics using machine learning

**DOI:** 10.3389/fpsyg.2026.1819513

**Published:** 2026-09-07

**Authors:** Gabriel Kadri, Raphael I. M. Santos, Felipe O. Aguiar, Victor H. O. Otani, Ricardo R. Uchida, Lucas M. Marques

**Affiliations:** 1 Mental Health Department, Santa Casa de São Paulo School of Medical Sciences, São Paulo, SP, Brazil; 2 Artificial Intelligence Research Division, Infinity Doctors Inc., Miami, FL, United States

**Keywords:** acoustics, biomarkers/behavioral markers, machine learning, psychology, sports, sports/e-sports performance, voice/speech acoustics

## Abstract

**Introduction:**

Competitive performance in electronic sports (e-sports) depends on rapid decision-making, emotional regulation, and coordinated communication, yet little is known about whether pre-match vocal behavior contains information predictive of competitive outcomes.

**Methods:**

This study applied supervised machine learning to 68 acoustic features extracted at the frame level (50-ms frames, 25-ms step) from 60-second pre-match team communication recordings in 89 professional Counter-Strike: Global Offensive matches; frame-level predictions were aggregated into a match-level score representing the proportion of frames classified as a win. Three predictive conditions were evaluated, acoustic features only, ranking difference only, and a combined model integrating both, using stratified group five-fold cross-validation. Uncertainty was quantified using percentile 95% confidence intervals from 2,000 match-level bootstrap resamples of the pooled out-of-fold predictions, with chance-level discrimination defined as AUC = 0.50.

**Results:**

Across algorithms, models combining voice and ranking achieved the strongest performance, with the Decision Tree classifier reaching a mean bootstrap AUC of 77.3% (95% CI 65.8–86.9) and accuracy of 78.6% (95% CI 69.9–86.7); all five voice-plus-ranking models had 95% CIs excluding chance. Voice-only models showed more limited evidence of above-chance discrimination: only the Decision Tree (AUC 67.8%, 95% CI 54.8–79.2) and Random Forest (AUC 64.0%, 95% CI 50.7–76.3) had confidence intervals excluding 0.50, whereas Linear Discriminant Analysis, Logistic Regression, and k-Nearest Neighbors did not. No ranking-only model showed a confidence interval excluding chance. Exploratory LIME-based feature-attribution analyses indicated that ranking difference received the highest within-model attribution in the combined models, while delta spectral flux, chroma standard deviation, and spectral centroid received the highest within-model attribution among acoustic descriptors for tree-based, linear, and distance-based classifiers, respectively; these rankings are descriptive and were not subjected to formal cross-model statistical comparison.

**Discussion:**

These findings provide preliminary, dataset-bounded evidence that acoustic patterns in brief pre-match team communication were associated with match outcome and, for some models, contributed predictive information beyond ranking; the retrospective, single-team design does not establish a generalizable behavioral biomarker or a causal link between vocal acoustics and competitive readiness.

## Introduction

The human voice is a multidimensional and highly sensitive behavioral signal capable of reflecting fluctuations in cognitive, emotional, and physiological states. Acoustic parameters such as fundamental frequency, spectral energy distribution, temporal stability, and Mel-frequency cepstral coefficients (MFCCs) consistently track changes in autonomic activation, psychomotor coordination, and affective expression (Cummins et al., 2015; Low et al., 2020; Veiga et al., 2025; Zhao et al., 2022). Because speech emerges naturally during communication and requires no external instrumentation, it offers an ecologically valid and continuously available window into internal processes, making it an increasingly valuable behavioral biomarker for real-world psychological assessment.

Competitive environments provide a particularly rich context for leveraging vocal behavior as an index of human performance. In these settings, individuals must regulate attention, manage emotional arousal, coordinate actions with others, and make rapid decisions under pressure—processes that directly influence engagement and cognitive workload, as demonstrated in studies using physiological metrics in gaming contexts (Ahmed et al., 2025). Prior research in psychophysiology shows that acute stress, heightened workload, and anticipatory arousal modulate attentional control and decision-making under pressure (Sharpe et al., 2024), reinforcing the relevance of behavioral markers capable of capturing these dynamics. These mechanisms are also known to shape vocal production through modulations in prosody, spectral characteristics, timing, and articulatory precision (Scherer, 2003). Empirical findings demonstrate that vocal parameters reliably encode fluctuations in stress, cognitive load, and emotional activation (Slavich et al., 2019; Veiga et al., 2025), supporting the use of acoustic analysis as an index of underlying psychophysiological processes.

Conceptually, anticipatory stress, cognitive workload, and arousal may influence respiratory support, laryngeal tension, articulatory precision, and prosodic control, thereby altering spectral, cepstral, energy, and timing-related voice measures. The same states may also be associated with attentional control, working-memory allocation, decision speed, and interpersonal coordination, which are central to competitive e-sports performance. Vocal acoustics may therefore function as an indirect correlate of performance-relevant cognitive–affective states. However, because these states were not directly measured and no mediation analysis was performed, the proposed pathway should be regarded as a conceptual framework rather than a demonstrated mechanism.

Professional electronic sports (e-sports) represent a unique and demanding example of such environments. Games like Counter-Strike: Global Offensive (CS:GO) require players to process complex sensory information, maintain situational awareness, demonstrate cognitive flexibility, and coordinate strategically with teammates in high-stakes, fast-paced situations. Studies of e-sports athletes highlight that cognitive functions such as working memory, perceptual-motor speed, and attentional shifting are critical determinants of performance (Sousa et al., 2020). Psychological factors such as anxiety, mental fatigue, and stress can impair these abilities, reducing precision, slowing reaction times, and undermining decision-making (Raglin, 2001; Tremayne and Barry, 2001), while simultaneously altering vocal characteristics in predictable ways (Cummins et al., 2015).

This convergence suggests that vocal expression may carry performance-relevant psychophysiological information in e-sports. Supporting this idea, our previous study using traditional regression analyses demonstrated that specific acoustic features, including tonal organization (Chroma_1_) and spectral variability (ΔMFCC_13_), were significantly associated with match outcomes in professional CS:GO, even after adjusting for contextual competitive factors such as team and opponent ranking. These results indicated that brief vocal samples collected before matches encode meaningful information about players' cognitive and emotional readiness, interpersonal coordination, and engagement under upcoming competitive demands. Our previous study addressed an explanatory question by estimating associations between selected acoustic descriptors and match outcome using conventional regression. The present study addresses a distinct predictive question: whether naturalistic pre-match communication can classify outcomes for held-out matches. It adds (i) grouped out-of-sample evaluation, (ii) a direct comparison of voice-only, ranking-only, and combined inputs, (iii) nonlinear learners evaluated alongside linear baselines, and (iv) *post hoc* examination of model-specific attributions. Accordingly, the contribution is predictive evaluation within this team's dataset, rather than a new demonstration that stress can alter the voice.

However, classical regression models are limited in their ability to capture the nonlinear and high-dimensional relationships that characterize naturalistic human communication. Machine learning approaches, by contrast, are specifically suited for modeling complex, multidimensional systems and identifying latent patterns not recoverable through linear methods (Berrar, 2019). Nonlinearity may matter because performance-relevant states need not have monotonic acoustic effects: moderate arousal may support engagement whereas excessive arousal may impair control, and the relevance of a spectral or prosodic change may depend simultaneously on energy, cepstral dynamics, or opponent-strength context. Tree-based and distance-based algorithms can represent thresholds, interactions, and locally varying decision regions, whereas Logistic Regression and Linear Discriminant Analysis provide linear benchmarks against which any benefit of additional model flexibility can be evaluated. Despite the rapid expansion of machine learning in behavioral, affective, and cognitive sciences, its application to voice-based prediction of competitive performance in e-sports remains scarce. Existing studies predicting e-sports outcomes have relied primarily on gameplay metrics or physiological sensors rather than vocal behavior (Lachowicz et al., 2024), leaving open the question of whether spontaneous pre-match communication can serve as a standalone predictive biomarker.

The present study addresses this gap by applying supervised machine learning algorithms to 68 acoustic features extracted from the first 60 s of pre-match team communication in professional CS:GO matches. This temporal window minimizes contamination from emotional reactions to in-game events and captures a psychologically meaningful preparatory phase characterized by anticipatory arousal, cognitive orientation, and early team coordination. To evaluate the relative contribution of communication-derived information, we compared models trained on acoustic features alone, models trained on ranking-based contextual information alone, and models trained on the combination of both. This design allows for a direct test of whether vocal expression contributes unique predictive information beyond established performance indicators.

Based on prior evidence linking vocal behavior to internal states (Cummins et al., 2015; Low et al., 2020; Veiga et al., 2025; Zhao et al., 2022) and performance-relevant psychological processes in competitive environments (Raglin, 2001; Tremayne and Barry, 2001), as well as findings from our previous regression-based study, we formulated two directional hypotheses evaluated at the match level. H1: models based exclusively on acoustic features would discriminate wins from losses above chance, operationalized as an out-of-fold AUC greater than 0.50. H2: for the same classification algorithm, combining acoustic features with the between-team ranking difference would yield a higher out-of-fold AUC than using ranking difference alone. AUC was the principal performance measure used to operationalize these hypotheses, calculated from the aggregated held-out match scores (the proportion of frames classified as a win within each recording). For H1, chance-level discrimination was defined as AUC = 0.50, and uncertainty was quantified using match-level bootstrap confidence intervals. For H2, the performance estimates of the combined and ranking-only conditions were compared descriptively using predictions obtained from the same held-out matches; because no paired inferential test of the between-condition AUC difference was conducted, these comparisons were not interpreted as formal evidence of statistical superiority. Feature-attribution analyses were exploratory.

By investigating whether pre-match vocal dynamics can accurately predict competitive outcomes in professional e-sports, this study advances a growing body of research emphasizing ecological validity in cognitive and affective assessment (Parsons, 2015). It also extends our previous work by moving from explanatory modeling to predictive analytics and contributes to the development of computational tools capable of capturing how communication, cognition, and emotion jointly shape human performance under pressure.

## Methods and analysis

### Design and dataset

This study used a retrospective observational design based on naturalistic audio recordings collected during professional Counter-Strike: Global Offensive (CS:GO) matches. The dataset comprised 89 official games played by a team of five professional athletes recruited from a competitive e-sports organization. All matches were conducted in ranked competitive settings following standard international CS:GO rules. For each game, only the first 60 s of team communication were analyzed. This temporal window was selected because it captures pre-engagement coordination and anticipatory cognitive–affective states while avoiding contamination from emotional reactions to in-game events, thus preserving the internal validity of the acoustic measures as indicators of preparatory processes. All procedures followed ethical guidelines for research with recorded speech and were approved by the institution responsible for the study.

### Audio recording and preprocessing

Audio was recorded through the team's standard communication system during live matches, using the players' original headsets. Recordings were stored in 44.1 kHz, 16-bit, single-channel WAV format, and no modifications were made to the athletes' microphones or communication setup. Because equipment quality and environmental conditions varied naturally across sessions—consistent with ecological studies of voice biomarkers—basic preprocessing steps were applied to improve signal quality while preserving authentic vocal characteristics. Segments with a duration of 100 ms or greater and an amplitude of less than −40 dB were removed to eliminate prolonged silences, and all files were normalized to allow comparison between games and speakers. After preprocessing, acoustic feature extraction was performed using the open-source pyAudioAnalysis library (Giannakopoulos, 2015), the same framework employed in our previous study to ensure methodological continuity.

A total of 68 acoustic features were extracted from each recording, encompassing time-domain, frequency-domain, and cepstral representations widely used in affective computing and speech analysis (Table 1). These included traditional prosodic descriptors (e.g., energy, zero-crossing rate), spectral measures (e.g., spectral centroid, spread, entropy, flux, and rolloff), harmonicity–noise indices, chroma-based tonal organization, and the full 13-coefficient MFCC vector along with its first-order deltas. For each 50-ms frame, pyAudioAnalysis version 0.3.14 computed 34 short-term acoustic descriptors. For descriptor j at frame t, the corresponding first-order delta feature was calculated as the signed frame-to-frame difference, Δx(j,t) = x(j,t) – x(j,t−1); consecutive frames had a 25-ms step and 50% overlap, the difference was not divided by the time interval, and no temporal regression or smoothing window was applied. The 34 delta values for the first frame were set to zero. Concatenating the 34 descriptors and their 34 delta features yielded 68 acoustic variables per frame. No feature-level pre-filtering, explicit feature selection, or dimensionality reduction was performed after extraction; all 68 acoustic variables were standardized and entered the voice-only models, and the combined models received the same 68 acoustic variables together with the ranking-difference variable. All features were z-score standardized prior to modeling. Because the dataset included repeated measures from the same group of players across multiple games, analyses were conducted at the match level, with the team's communication considered as a single multifeature acoustic sample representing the collective pre-match vocal profile. For each match, one 60-s single-channel recording captured the team's collective communication during the warm-up and preparation period immediately before the match. The recording contained the group discussion as a whole; individual players were not recorded on separate channels and no speaker diarization was performed. Segments lasting at least 100 ms with amplitude below −40 dB were removed as prolonged silence or low-amplitude segments, and the remaining audio was normalized. Each processed recording was segmented into 50-ms frames using a 25-ms step (50% overlap), and 68 acoustic variables were extracted per frame. All frames inherited the outcome label of their match. Five-fold StratifiedGroupKFold cross-validation used the recording identifier as the grouping variable, ensuring that frames from the same match were assigned to the same fold, and models were trained at the frame level. For a held-out match m containing n_m frames, the match score was calculated as s_m = (1/n_m) Σ_*i*_ ŷ_mi, where ŷ_mi ϵ {0,1} was the binary prediction for frame i; s_m therefore represented the proportion of frames classified as a victory, and match-level classifications and performance measures were derived from this aggregated score. We note that our originally submitted description of one acoustic feature vector per match was inaccurate: feature extraction and model fitting occurred at the frame level, whereas predictions were aggregated at the match level.

**Table 1:** Acoustic feature set used in the machine learning models.

Index	Name	Description
1	Zero crossing rate	The rate of sign-changes of the signal during the duration of a particular frame.
2	Energy	The sum of squares of the signal values, normalized by the length.
3	Entropy of energy	The entropy of sub-frames normalized energies. It can be interpreted as a measure of abrupt changes.
4	Spectral centroid	The center of gravity of the spectrum.
5	Spectral spread	The second of gravity of the spectrum.
6	Spectral entropy	Entropy of the normalized spectral energies for a set of sub-frames
7	Spectral flux	The squared difference between the normalized magnitudes of the spectra of the two successive frames.
8	Spectral rolloff	The frequency below which 90% of the magnitude distribution of the spectrum is concentrated.
9–21	MFCCs	Mel Frequency Cepstral Coefficients form a cepstral representation where the frequency bands are not linear but distributed according to the mel-scale.
22–33	Chroma vector	A 12-element representation of the spectral energy where the bins represent the 12 equal-tempered pitch classes of western-type music (semitone spacing).
34	Chroma deviation	The standard deviation of the 12 chroma coefficients.

The table lists the 34 short-term acoustic descriptors computed using pyAudioAnalysis version 0.3.14, following the feature set described by Giannakopoulos (2015). Each descriptor was accompanied by a first-order delta calculated as the signed difference between consecutive frames, whose starting positions were separated by a 25-ms step. Delta values for the first frame were set to zero. The 34 descriptors and their 34 corresponding deltas yielded 68 acoustic variables per frame. No feature-level pre-filtering, explicit feature-selection procedure, or dimensionality reduction was applied.

### Predictive variables and modeling conditions

To evaluate predictive capacity, match outcomes (win or loss) served as the dependent variable. Ranking differences between the team and its opponents, derived from the official competitive ranking system used by the league, were included as contextual performance indicators. Three modeling conditions were created to assess the relative and combined contributions of communication-derived and contextual information: (i) models trained exclusively on acoustic features; (ii) models trained exclusively on ranking information, and (iii) models trained on both acoustic and ranking inputs. This structure allowed us to quantify the incremental predictive value of vocal behavior beyond established performance metrics.

### Machine learning models and optimization

Supervised machine learning models were implemented in Python using scikit-learn (Pedregosa et al., 2011). Five algorithms were selected to represent a diverse range of decision functions and modeling assumptions: Logistic Regression, Linear Discriminant Analysis, k-Nearest Neighbors, Decision Tree, and Random Forest. These models span linear, distance-based, and ensemble-based approaches, enabling evaluation of nonlinear and interaction-driven predictive patterns. To mitigate overfitting and ensure stability, hyperparameter optimization was performed using Tree-Structured Parzen Estimation within the Optuna framework (Akiba et al., 2019). Hyperparameters were optimized separately for each algorithm and outer training fold using Optuna's seeded Tree-Structured Parzen Estimator sampler (seed = 42; 50 trials; direction = maximize). The objective was the mean macro-F1 score across inner stratified grouped folds. After selection, each model was refitted on the complete outer training fold and evaluated once on the held-out matches. Complete search spaces and fold-specific selected values are reported in Supplementary Tables S1-A, S1-B, and S1-C. Because the dataset exhibited modest class imbalance, undersampling through the NearMiss algorithm was applied within the training folds to balance class representation while preserving the natural distribution in the test sets. After the scaler was fitted exclusively to the training data, NearMiss was applied only to the training frames; held-out frames retained their original class distribution. The implementation used imbalanced-learn version 0.12.3 with the default parameters sampling_strategy = “auto”, version = 1, and n_neighbors = 3. For this binary classification task, the majority class was reduced to the number of observations in the minority class, with majority-class frames selected according to their mean distance to the three nearest minority-class frames. NearMiss was chosen to reduce frame-level imbalance while retaining observed acoustic frames and avoiding the generation of synthetic observations from highly overlapping and correlated frames. This choice nonetheless represents a modeling limitation: because NearMiss preferentially selects majority-class observations close to minority-class observations, it may be sensitive to noise, outliers, or class overlap and may discard informative majority-class frames. We did not compare NearMiss with class weighting, random undersampling, or training without resampling, so the extent to which the reported results depend on the selected imbalance-handling strategy remains unknown.

Model performance was evaluated using stratified group k-fold cross-validation with five folds, ensuring that matches from the same competitive series appeared only in either training or test sets, thus preventing information leakage across folds. Standard predictive metrics were computed, including accuracy, recall, specificity, F1-score, and the area under the receiver operating characteristic curve (AUC).

To interpret the decision mechanisms of the best-performing models, local feature attribution was examined using the Local Interpretable Model-Agnostic Explanation (LIME) framework (Ribeiro et al., 2016), which generates instance-level explanations by approximating each model's decision boundary with an interpretable linear surrogate. This approach enabled identification of the features most influential in individual predictions and facilitated comparison across algorithms regarding their reliance on acoustic or contextual information. Feature attribution was examined using LIME version 0.2.0.1 for all classifiers. For each model and cross-validation fold, LIME generated local explanations for up to 100 held-out frames. The absolute attribution weights were averaged by feature across the explained instances and subsequently aggregated across the five folds, and features were then ranked within each model according to their mean absolute LIME attribution. Native model-specific importance measures, such as impurity-based importance for tree models or coefficient magnitudes for linear models, were not used to construct Table 2. This aggregation did not preserve the direction of individual feature contributions or their association with specific frames, and instance-linked signed explanations were not retained; the resulting rankings therefore summarize the analyzed frame-level explanations and do not constitute instance-level explanations of individual predictions or direct explanations of the aggregated match-level classification. LIME was used exclusively for exploratory *post hoc* interpretation and did not select variables during model training.

**Table 2:** Mean and standard deviation of performance across the five folds.

Voice + Ranking							
Models	**Recall (%)**	**Specificity (%)**	**PPV (%)**	**NPV (%)**	**Accuracy (%)**	**F1-score (%)**	**AUC (%)**
**DT**	**71.4 (16.5)**	**84.6 (23.4)**	**79.7 (30.0)**	**82.7 (9.4)**	**76.1 (12.7)**	**68.6 (14.3)**	**78.0 (4.9)**
LDA	88.6 (6.0)	59.9 (20.8)	58.5 (21.5)	87.9 (7.2)	69.0 (13.9)	67.7 (15.7)	74.2 (9.5)
LR	88.6 (6.0)	59.9 (20.8)	58.5 (21.5)	87.9 (7.2)	69.0 (13.9)	67.7 (15.7)	74.2 (9.5)
kNC	63.8 (21.0)	73.5 (18.4)	62.2 (11.0)	79.7 (6.2)	72.3 (6.3)	61.1 (11.5)	68.6 (3.5)
15.6-7.4,-1.3498ptRF	74.3 (14.7)	81.3 (12.0)	70.4 (15.4)	81.9 (10.1)	77.1 (8.4)	69.9 (6.2)	77.8 (8.4)
Voice							
DT	74.3 (33.5)	62.2 (25.7)	62.0 (25.7)	84.7 (15.6)	63.9 (10.2)	55.4 (20.3)	68.3 (6.5)
LDA	**71.4 (21.3)**	**70.3 (8.6)**	**57.8 (13.2)**	**80.0 (13.0)**	**68.7 (4.7)**	**60.6 (10.2)**	**70.9 (6.7)**
LR	54.3 (34.9)	80.6 (13.5)	48.3 (28.7)	74.7 (15.3)	67.3 (5.9)	47.4 (25.5)	67.5 (10.9)
kNC	56.2 (35.3)	68.7 (32.6)	43.2 (23.9)	78.0 (17.4)	63.7 (12.7)	46.9 (25.4)	62.4 (10.2)
15.6-7.4,-1.3498ptRF	77.1 (15.3)	60.8 (12.3)	53.9 (10.4)	79.3 (12.8)	66.1 (6.1)	61.6 (5.5)	69.0 (8.7)
Ranking							
DT	62.8 (22.5)	58.4 (16.1)	47.1 (14.5)	73.0 (15.5)	56.8 (10.2)	50.1 (11.1)	60.6 (6.9)
LDA	**52.4 (27.1)**	**61.7 (16.7)**	**46.8 (24.9)**	**71.7 (16.4)**	**58.2 (15.5)**	**46.9 (23.6)**	**57.0 (17.0)**
**LR**	**52.4 (27.1)**	**61.7 (16.7)**	**46.8 (24.9)**	**71.7 (16.4)**	**58.2 (15.5)**	**46.9 (23.6)**	**57.0 (17.0)**
kNC	39.0 (15.7)	55.8 (15.6)	34.6 (12.1)	60.1 (13.5)	47.1 (8.2)	33.6 (7.2)	47.4 (7.4)
RF	60 (25.9)	54.4 (17.7)	43.5 (17.3)	69.9 (19.3)	53.2 (13.7)	46.8 (14.9)	57.2 (13.0)

The values represent the average performance (SD) obtained in a group-stratified five-fold cross-validation. All values are expressed as percentages. All models were trained in three ways: (i) using the 68 acoustic features extracted from a 60-second segment of team communication prior to the start of the match, incorporating ranking information; (ii) using only the 68 acoustic features; and (iii) using only ranking information. The metrics are defined as follows: Accuracy = proportion of correctly classified matches; Recall = sensitivity, or proportion of correctly identified wins; Specificity = proportion of correctly identified losses; PPV = positive predictive value; NPV = negative predictive value; F1-score = harmonic mean of precision and recall; AUC = area under the receiver operating characteristic (ROC) curve. Bold values indicate the highest mean absolute LIME attribution within each model. Bold values indicate the highest mean absolute LIME attribution within each model.

All analyses were conducted to maintain coherence with our previous regression-based investigation of vocal predictors of competitive performance while extending that work into predictive modeling using nonlinear, high-dimensional machine learning methods. This methodological framework allowed us to rigorously assess whether pre-match vocal expression contains sufficient psychophysiological and communicative information to forecast competitive outcomes in professional e-sports.

## Results

### Descriptive overview of the dataset

The final dataset comprised 89 professional Counter-Strike: Global Offensive (CS:GO) matches played by a team of five elite athletes. For each match, a 60-s segment of pre-match team communication was analyzed, resulting in frame-level acoustic feature vectors (50-ms frames, 25-ms step) that were subsequently aggregated into a single match-level prediction score, as detailed in the Methods. A total of 68 acoustic features were extracted from each recording, and ranking difference between the team and its opponent served as the contextual performance indicator. The dataset exhibited modest class imbalance, with a slightly higher proportion of victories than defeats, and variability across matches reflected natural fluctuations in team coordination, opponent strength, and competitive context. All models were evaluated under identical cross-validation constraints to ensure comparability of results and avoid information leakage.

## Model performance using voice and ranking features

When acoustic features and ranking differences were combined, all machine learning models achieved their strongest predictive performance (Table 3). The Decision Tree classifier demonstrated the most balanced and robust results, with mean accuracy of 76.1 (SD = 12.7), recall of 71.4 (16.5), specificity of 84.6 (23.4), positive predictive value (PPV) of 79.7 (30.0), negative predictive value (NPV) of 82.7 (9.4), F1-score of 68.6 (14.3), and an AUC of 78.0 (4.9). The Random Forest classifier performed similarly, with accuracy of 77.1 (8.4), recall of 74.3 (14.7), specificity of 81.3 (12.0), PPV of 70.4 (15.4), NPV of 81.9 (10.1), F1-score of 69.9 (6.2), and AUC of 77.8 (8.4). Linear Discriminant Analysis and Logistic Regression converged on identical performance metrics within this configuration, yielding accuracy of 69.0 (13.9), recall of 88.6 (6.0), specificity of 59.9 (20.8), PPV of 58.5 (21.5), NPV of 87.9 (7.2), F1-score of 67.7 (15.7), and AUC of 74.2 (9.5). We verified that these identical values occurred at the aggregated match-classification level in the ranking-only and combined conditions, but not in the voice-only condition. Inspection of the continuous frame-level scores and fitted parameters showed that the two models were not identical; rather, aggregation and thresholding resulted in the same binary assignments for the held-out matches, and in the ranking-only condition both models operated on a single standardized predictor and produced the same ordering after thresholding. These identical summary metrics should therefore not be interpreted as evidence of identical decision boundaries or complete linear separability. The k-Nearest Neighbors model produced accuracy of 72.3 (6.3), recall of 63.8 (21.0), specificity of 73.5 (18.4), PPV of 62.2 (11.0), NPV of 79.7 (6.2), F1-score of 61.1 (11.5), and AUC of 68.6 (3.5). Overall, the joint inclusion of vocal and ranking cues consistently produced descriptively superior predictive performance across algorithms. Because no paired inferential test of the between-condition AUC difference was conducted, this comparison is descriptive and is not presented as formal evidence of statistical superiority (see below for the bootstrap-based chance-level analysis, which provides the inferential evidence reported in this study). All values in Table 3 are expressed as percentages and summarize the mean and standard deviation across the five cross-validation folds.

**Table 3:** Top ten most influential predictors for each machine learning model based on feature-importance rankings.

Feature ranking	Models				
	DT	kNC	LDA	LR	RF
1	Delta ranking	Delta ranking	Delta ranking	Delta ranking	Delta ranking
2	Delta spectral flux	Spectral cntroid	Chroma std	Chroma std	Delta spectral flux
3	Delta MFCC 7	Spectral entropy	Energy	Chroma 7	Spectral flux
4	Energy entropy	MFCC 2	Chroma 7	Energy	Delta MFCC 1
5	Delta chroma std	MFCC 3	Chroma 3	Chroma 3	Delta MFCC 2
6	Delta MFCC 11	MFCC 6	MFCC 2	MFCC 2	Energy entropy
7	ZCR	Spectral spread	MFCC 7	Spectral spread	Delta MFCC 3
8	Delta MFCC 1	ZCR	Spectral spread	MFCC 7	Delta MFCC 4
9	Spectral centroid	Chroma 5	Spectral rolloff	Spectral entropy	Chroma 1
10	Delta MFCC 3	Spectral rolloff	Chroma 1	Chroma 5	Delta energy entropy

The table presents the ten highest-ranked predictors for each classifier when trained using acoustic features and rank-difference information (Delta Ranking), ordered from highest to lowest mean absolute LIME attribution within each model. DT = Decision Tree; kNC = k-Nearest Neighbors Classifier; LDA = Linear Discriminant Analysis; LR = Logistic Regression; RF = Random Forest. All rankings were generated using the same model-agnostic LIME procedure (mean absolute local attribution aggregated across up to 100 held-out frames per fold and across the five folds); native, model-specific importance measures (e.g., impurity reduction for tree-based models, coefficient magnitude for linear models) were not used to construct this table. Because LIME approximates each fitted model locally and its output depends on the perturbation procedure and analyzed instances, rankings are not directly comparable across models.

## Model performance using voice features only

Models trained exclusively on acoustic features exhibited reduced but clearly above-chance predictive performance (Table 3). Linear Discriminant Analysis showed the strongest performance in this condition, with mean accuracy of 68.7 (4.7), recall of 71.4 (21.3), specificity of 70.3 (8.6), PPV of 57.8 (13.2), NPV of 80.0 (13.0), F1-score of 60.6 (10.2), and an AUC of 70.9 (6.7). The Decision Tree classifier yielded accuracy of 63.9 (10.2), recall of 74.3 (33.5), specificity of 62.2 (25.7), PPV of 62.0 (25.7), NPV of 84.7 (15.6), F1-score of 55.4 (20.3), and an AUC of 68.3 (6.5). Logistic Regression achieved accuracy of 67.3 (5.9), recall of 54.3 (34.9), specificity of 80.6 (13.5), PPV of 48.3 (28.7), NPV of 74.7 (15.3), F1-score of 47.4 (25.5), and an AUC of 67.5 (10.9). k-Nearest Neighbors produced accuracy of 63.7 (12.7), recall of 56.2 (35.3), specificity of 68.7 (32.6), PPV of 43.2 (23.9), NPV of 78.0 (17.4), F1-score of 46.9 (25.4), and AUC of 62.4 (10.2). Random Forest obtained accuracy of 66.1 (6.1), recall of 77.1 (15.3), specificity of 60.8 (12.3), PPV of 53.9 (10.4), NPV of 79.3 (12.8), F1-score of 61.6 (5.5), and AUC of 69.0 (8.7). These fold-level descriptive results suggested that short segments of pre-match communication contain acoustic information relevant to match outcome, even without contextual performance indicators; as detailed in the Chance-Level Comparisons subsection below, however, only the Decision Tree and Random Forest voice-only models showed bootstrap confidence intervals that excluded chance, so this evidence should not be generalized to all five voice-only classifiers.

## Model performance using ranking features only

Ranking-only models showed the lowest predictive performance across all metrics (Table 2). The Decision Tree classifier reached accuracy of 56.8 (10.2), recall of 62.8 (22.5), specificity of 58.4 (16.1), PPV of 47.1 (14.5), NPV of 73.0 (15.5), F1-score of 50.1 (11.1), and AUC of 60.6 (6.9). Linear Discriminant Analysis and Logistic Regression again produced identical metrics, with accuracy of 58.2 (15.5), recall of 52.4 (27.1), specificity of 61.7 (16.7), PPV of 46.8 (24.9), NPV of 71.7 (16.4), F1-score of 46.9 (23.6), and AUC of 57.0 (17.0). The poorest performance was observed in the k-Nearest Neighbors model, with accuracy of 47.1 (8.2), recall of 39.0 (15.7), specificity of 55.8 (15.6), PPV of 34.6 (12.1), NPV of 60.1 (13.5), F1-score of 33.6 (7.2), and AUC of 47.4 (7.4). Random Forest produced accuracy of 53.2 (13.7), recall of 60.0 (25.9), specificity of 54.4 (17.7), PPV of 43.5 (17.3), NPV of 69.9 (19.3), F1-score of 46.8 (14.9), and AUC of 57.2 (13.0). The comparatively weak performance of ranking-only models underscores the added value of pre-match vocal behavior as a candidate predictor of competitive readiness.

## Chance-level comparisons

**Figure 1:**
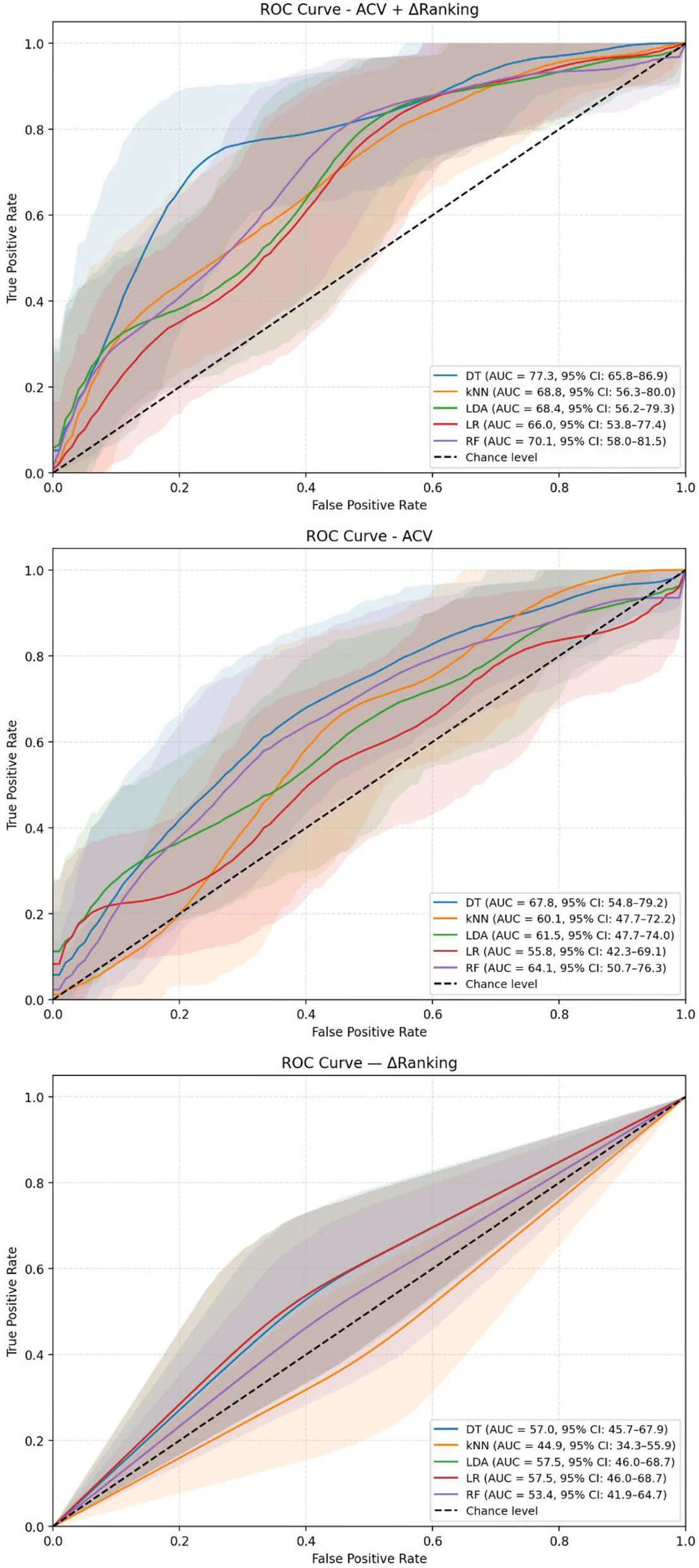
Receiver operating characteristic curves for the five classifiers under three prediction conditions: **(A)** voice plus Δranking, **(B)** voice only, and **(C)** Δranking only. Solid lines represent the mean ROC curves obtained from 2,000 bootstrap resamples of the pooled out-of-fold match predictions, and shaded regions represent pointwise percentile 95% confidence intervals. The legends report the mean bootstrap AUC and its percentile 95% confidence interval. The dashed diagonal represents chance-level discrimination (AUC = 0.50).

Chance-level discrimination was defined as AUC = 0.50. Uncertainty in the out-of-fold AUC estimates was quantified using 2,000 bootstrap resamples of the pooled match-level predictions, with the match/audio recording (rather than the individual acoustic frame) used as the resampling unit; resamples containing only one outcome class were discarded, and percentile 95% confidence intervals were defined by the 2.5th and 97.5th percentiles of the valid bootstrap distribution (Table 4; Figure 1). Evidence of above-chance discrimination was considered present only when the lower bound of the 95% confidence interval exceeded 0.50. All five voice-plus-ranking models met this criterion. Among the voice-only models, the Decision Tree (AUC = 0.678, 95% CI 0.548–0.792) and Random Forest (AUC = 0.641, 95% CI 0.507–0.763) showed evidence of above-chance discrimination, whereas the confidence intervals for kNN (AUC = 0.601, 95% CI 0.477–0.722), Linear Discriminant Analysis (AUC = 0.615, 95% CI 0.477–0.740), and Logistic Regression (AUC = 0.558, 95% CI 0.423–0.691) included 0.50 and therefore did not support an above-chance interpretation. We accordingly removed our previous, unsupported statement that all voice-only classifiers exceeded chance. No ranking-only model's confidence interval excluded 0.50.

**Table 4:** Bootstrap estimates and percentile 95% confidence intervals for the pooled out-of-fold match-level performance.

Voice + Δ Ranking							
Models	**Recall (%)**	**Specificity (%)**	**PPV (%)**	**NPV (%)**	**Accuracy (%)**	**F1-score (%)**	**AUC (%)**
**DT (IC95%)**	**77.3 (62.1–91.3)**	**79.4 (67.3–90.0)**	**69.1 (52.9–84.2)**	**85.6 (75.0–94.6)**	**78.6 (69.9–86.7)**	**72.6 (60.0–83.1)**	**77.3 (65.8–86.9)**
LDA (IC95%)	81.0 (36.4 – 96.8)	58.4 (38.9 – 96.1)	55.3 (39.1 – 86.7)	85.2 (68.1 – 96.7)	68.4 (56.2 – 79.3)	63.7 (48.0 – 75.4)	68.4 (56.2 – 79.3)
LR (IC95%)	83.0 (44.7 – 100)	54.1 (33.9 – 88.5)	52.4 (38.0 – 71.11)	85.3 (69.6 – 100)	64.8 (53.0 – 74.7)	63.2 (49.0 – 74.7)	66.0 (53.8 – 77.4)
kNC (IC95%)	69.5 (32.5 – 97.2)	65.4 (27.9 – 96.1)	58.0 (37.9 – 86.7)	80.5 (64.9 – 96.8)	66.9 (51.8 – 78.3)	59.9 (44.4 – 72.3)	68.8 (56.3 – 80.0)
RF 15.6-7.2,-1.3499pt(IC95%)	81.5 (42.3 – 96.5)	61.0 (43.8 – 93.3)	56.2 (40.9 – 78.1)	85.5 (71.4 – 96.8)	68.7 (57.8 – 78.3)	65.4 (50.8 – 76.9)	70.1 (58.0 – 81.5)
Voice							
**DT (IC95%)**	**69.4 (38.7–94.6)**	**65.8 (34.5–89.8)**	**56.1 (38.2–76.5)**	**79.4 (65.8–93.5)**	**67.1 (53.0–78.3)**	**60.4 (44.4–72.7)**	**67.8 (54.8–79.2)**
LDA (IC95%)	57.8 (18.2 – 96.5)	69.1 (25.0 – 98.2)	59.3 (36.3 – 92.3)	75.6 (61.2 – 94.1)	64.9 (48.2 – 77.1)	52.7 (28.6 – 68.2)	61.5 (47.7 – 74.0)
LR (IC95%)	55.6 (14.7 – 93.9)	67.3 (28.0 – 100)	59.6 (34.7 – 100)	74.5 (59.4 – 91.7)	62.9 (48.2 – 77.1)	49.3 (24.4 – 67.3)	55.8 (42.3 – 69.1)
kNC (IC95%)	77.3 (56.2 – 100)	53.2 (20.0 – 74.0)	50.1 (35.0 – 65.7)	81.1 (66.7 – 100)	62.1 (47.0 – 73.5)	59.9 (47.6 – 72.0)	60.1 (47.7 – 72.2)
RF 15.6-7.2,-1.3499pt(IC95%)	65.1 (32.3 – 90.9)	67.5 (38.0 – 92.6)	55.9 (38.1 – 77.1)	77.4 (64.2 – 91.2)	66.7 (54.2 – 77.1)	58.4 (41.5 – 71.0)	64.0 (50.7 – 76.3)
Δ**Ranking**							
DT (IC95%)	58.0 (39.1 – 75.0)	56.1 (42.5 – 69.8)	43.8 (27.5 – 59.0)	69.3 (55.0 – 82.6)	56.8 (45.8 – 67.5)	49.6 (33.3 – 62.8)	57.0 (45.7 – 67.9)
**LDA (IC95%)**	**55.1 (36.7–72.7)**	**60.0 (46.3–73.1)**	**44.89 (28.9–61.7)**	**69.3 (55.3–82.2)**	**58.1 (47.0–68.7)**	**49.1 (33.9–63.6)**	**57.5 (46.0–68.74)**
**LR (IC95%)**	**55.1 (36.7–72.7)**	**60.0 (46.3–73.1)**	**44.89 (28.9– 61.7)**	**69.3 (55.3–82.2)**	**58.1 (47.0–68.7)**	**49.1 (33.9–63.6)**	**57.5 (46.0–68.74)**
kNC (IC95%)	35.7 (18.9 – 52.9)	54.1 (40.4 – 68.5)	31.5 (16.1 – 47.4)	58.7 (44.9 – 72.1)	47.3 (36.1 – 57.8)	33.1 (17.8 – 47.1)	44.9 (34.2 – 55.9)
RF (IC95%)	54.6 (36.3 – 71.9)	52.1 (38.8 – 66.0)	40.3 (24.4 – 55.3)	66.0 (51.3 – 79.5)	53.1 (42.2 – 63.8)	46.0 (30.5 – 59.7)	53.4 (41.9 – 64.7)

Values are presented as the mean bootstrap estimate (percentile 95% confidence interval) obtained from 2,000 resamples of the pooled out-of-fold match predictions. The match/audio recording was used as the resampling unit, and resamples containing only one outcome class were discarded. The global bootstrap analysis used random seed 142. Bold values indicate models showing evidence of above-chance discrimination, defined as a 95% bootstrap confidence interval for the AUC with a lower bound greater than 0.50.

## Feature importance across algorithms

Feature importance analyses revealed both convergence and algorithm-specific patterns. Table 2 presents the ten features with the highest mean absolute LIME attribution within each model; native model-specific importance measures, such as impurity-based importance for tree models or coefficient magnitudes for linear models, were not used to construct this table. Ranking difference occupied the first position within each of the five combined models. Among the acoustic variables, delta spectral flux received the highest within-model position for the Decision Tree and Random Forest models, whereas spectral centroid, chroma standard deviation, and other descriptors occupied the leading acoustic positions in the remaining models. These rankings describe how LIME approximated the behavior of each fitted classifier and were not subjected to formal statistical comparisons between models; the recurrence of individual descriptors across models is therefore reported only as an exploratory descriptive observation and should not be interpreted as evidence of convergence, causal relevance, or a shared latent psychophysiological signal.

Across all models, several acoustic descriptors repeatedly appeared among the top LIME-attributed contributors, including energy entropy, MFCC coefficients (notably MFCC2, MFCC3, MFCC6, and MFCC7), their corresponding deltas (ΔMFCC1, ΔMFCC2, ΔMFCC3, ΔMFCC4, and ΔMFCC7), zero-crossing rate, spectral spread, spectral rolloff, spectral entropy, and specific chroma components (Chroma 1, Chroma 3, Chroma 5, and Chroma 7). These descriptive patterns are consistent with predictive information being distributed across multiple acoustic dimensions rather than concentrated in a single feature, but—consistent with the exploratory, model-agnostic nature of the LIME procedure described above—we no longer interpret this as evidence that team communication “encodes” shared psychophysiological signatures; the attributions describe how each fitted model behaved on the analyzed frames rather than a validated psychological mechanism.

## Discussion

The present study demonstrates that short segments of pre-match team communication contain structured acoustic signals that meaningfully contribute to forecasting competitive outcomes in professional e-sports. As hypothesized, models integrating vocal features with ranking differences consistently outperformed those relying on a single modality, confirming that vocal behavior provides information that is complementary—but not redundant—to contextual competitive indicators. Ranking difference remained the strongest isolated predictor, consistent with its structural role in shaping match expectations. Yet the substantial performance gains observed when acoustic features were added indicate that pre-match vocal expression captures emergent cognitive–affective and interpersonal dynamics not accounted for by ranking alone. These findings align with contemporary perspectives on speech as a multidimensional biosignal that was associated with psychophysiological states relevant to performance (Slavich et al., 2019).

Importantly, voice-only models also achieved above-chance accuracy and AUC values, despite relying on just 60 s of spontaneous communication recorded in naturalistic, high-stakes conditions. This supports our hypothesis that unscripted vocal exchanges may correspond to markers of readiness, attentional engagement, affective regulation, and early team coordination. Prior research demonstrates that acoustic parameters—including spectral energy distribution, MFCC variability, and prosodic modulation—systematically index fluctuations in arousal, cognitive workload, and emotional activation (Veiga et al., 2025). The current results extend this literature by showing that such vocal patterns do not merely correlate with internal states but also possess prospective predictive value when analyzed using nonlinear, high-dimensional machine learning models.

The pattern of LIME attributions across models is reported descriptively rather than as evidence of a shared underlying signal. Tree-based algorithms emphasized delta spectral flux—capturing rapid fluctuations in spectral energy—which is plausibly consistent with, though not evidence of, sensitivity to fine-grained, dynamic modulation associated with arousal and cognitive load (Slavich et al., 2019). Linear classifiers prioritized chroma variability, indicative of tonal organization and prosodic stability, whereas distance-based classification relied heavily on spectral centroid, corresponding to shifts in average frequency distribution. This pattern is consistent with predictive information being distributed across combinations of prosodic, spectral, cepstral, and tonal descriptors rather than concentrated in a single marker, in line with prior work in computational paralinguistics and affective voice modeling (Schuller and Batliner, 2013). We emphasize, however, that these LIME-based rankings should not be interpreted as evidence of convergence, causal relevance, or a shared latent psychophysiological signal across algorithms: LIME provides local approximations of model behavior, and its attribution values depend on the fitted model, the analyzed instances, and the perturbation procedure. Differences and similarities across models are therefore reported as exploratory, hypothesis-generating observations rather than a demonstration that diverse acoustic pathways access a common signal relevant to pre-performance states.

The interpretability analysis does not eliminate the black-box limitation of these models. LIME approximates a model locally around a selected observation, and its explanation depends on the perturbation distribution, neighborhood definition, and local surrogate fidelity; correlated acoustic descriptors may receive unstable or interchangeable weights, and averaging absolute local contributions can obscure heterogeneity across matches and outcome classes. Furthermore, LIME was applied to frame-level predictions, whereas the final match classification was obtained by aggregating predictions across frames, so the reported rankings do not directly explain the final match-level decision, and instance-linked signed explanations were not retained, preventing evaluation of the coherence and direction of the contributions associated with any specific prediction. Accordingly, the reported attributions are hypothesis-generating descriptions of model behavior; they do not identify causal vocal mechanisms, validate latent psychological states, or constitute instance-level explanations of individual predictions. Future studies should preserve instance-linked explanations and align the interpretability procedure with the final unit of prediction.

Taken together, these results support the view that pre-match communication is not merely functional information exchange but also a behavioral substrate carrying subtle signatures of team-level readiness, interpersonal attunement, and shared mental models (Santos et al., 2016). In e-sports, where coordination, situational awareness, and rapid decision-making are critical for performance, vocal expression appears to serve as an emergent indicator of cognitive–affective preparedness. The present findings also complement recent evidence showing that cognitive and attentional performance in e-athletes can be modulated by training experiences and environmental cues (Lachowicz et al., 2024), suggesting that vocal behavior may correspond to dynamic performance-relevant states within these competitive settings.

## Interpretation boundaries and implications for future research

Although the combined models showed AUC confidence intervals that excluded chance and voice-only evidence was more limited (present only for the Decision Tree and Random Forest), these predictive effects must be interpreted within the limits of the dataset. Matches were drawn from a single professional team, and vocal patterns may be associated with team-specific communication styles, tactical routines, or interpersonal norms. While stratified group cross-validation mitigates some risk of overfitting, generalizability across teams, competitive tiers, and game genres remains an open question. Additionally, only pre-match communication was analyzed; vocal signatures likely evolve across the match as players experience fluctuating cognitive load, emotional intensity, and tactical transitions. Research indicates that vocal parameters change dynamically in response to increasing task difficulty and stress (Veiga et al., 2025), suggesting that longitudinal voice tracking across entire matches could uncover additional predictive structures.

Another interpretational boundary concerns the nature of the predictive signal itself. Although above-chance forecasting was achieved, these accuracy levels do not imply deterministic relationships between vocal expression and match outcome nor identify causal mechanisms. Vocal features likely capture a constellation of interacting factors—including psychophysiological readiness, attentional orientation, emotional regulation, coordination efficacy, and shared situational models—but the present design cannot disentangle their relative contributions. Integrating additional modalities such as physiological sensing, linguistic content, gameplay telemetry, or team cohesion measures may clarify these pathways. Multimodal approaches consistently outperform unimodal prediction in behavioral science and affective computing (Rovelli et al., 2025), and future work may benefit from embedding vocal analytics within broader computational pipelines.

Despite these limitations, the findings open promising avenues for performance science in e-sports. Vocal analytics may offer a low-cost, noninvasive method for monitoring readiness and interpersonal alignment in both training and competition environments. A possible future use, after prospective and external validation, would be a training-only decision-support dashboard that analyzes a standardized 60-second warm-up sample and compares the team-level score with that team's longitudinal baseline. A marked, low-confidence deviation could prompt a voluntary check-in about fatigue, communication quality, or technical conditions before practice. It should not automatically select lineups, discipline players, or infer a mental disorder. Any deployment would require informed consent, restricted access, transparent communication of uncertainty, data-minimization safeguards, and evaluation of whether the alert improves decisions rather than merely predicting past outcomes. The results also underscore the potential of computational voice models to serve as digital biomarkers within emerging frameworks of digital phenotyping and precision performance monitoring. Finally, the methodological framework developed here demonstrates that ecologically valid, high-dimensional vocal data—even when brief and unscripted—can be leveraged by machine learning to forecast performance-relevant states, providing an alternative to traditional laboratory-based paradigms that may lack ecological fidelity.

## Conclusions

This study provides evidence that, within this retrospective dataset from one professional team, pre-match vocal behavior carried acoustic information that contributed to out-of-fold prediction of match outcome in professional e-sports, particularly when combined with contextual indicators such as ranking difference. These findings support further investigation of team voice acoustics as a candidate performance-related signal; they do not establish a generalizable biomarker of readiness, coordination, or cognitive–affective state, and the current accuracy estimates may be optimistic pending nested-fold hyperparameter validation and external replication. More broadly, the study bridges explanatory and predictive approaches to human communication, illustrating how nonlinear models can uncover latent patterns in naturalistic speech that traditional statistical methods may overlook. Future studies should preregister the analysis and primary metric; recruit larger samples from multiple teams, tournaments, competitive levels, and game genres; use nested group-aware validation followed by external temporal and team-level testing; standardize microphone and sampling conditions; compare pre-match with defined early- and late-match windows; separate speakers where feasible; and evaluate whether linguistic, gameplay, physiological, and cohesion measures add incremental value. Interpretability should be assessed for stability and fidelity, and prospective utility studies should examine calibration, fairness, privacy, player consent, and the consequences of false alerts before any operational use.

## Data Availability

The raw data supporting the conclusions of this article will be made available by the authors, without undue reservation.
