# Supplementary material for: Predicting competitive outcomes in professional e-sports from 60-second pre-match voice acoustics using machine learning
Source: Front Psychol. 2026 Sep 7;17:1819513. doi: 10.3389/fpsyg.2026.1819513 (PMC13590748; doi:10.3389/fpsyg.2026.1819513)
Supplement: Supplementary file 1 [file Data_Sheet_1.pdf]

Table S1-A - Hyperparameter optimization of models trained solely on ranking differences.

Model	Space Parameters	Fold 1 Best Parameters	Fold 2 Best Parameters	Fold 3 Best Parameters	Fold 4 Best Parameters	Fold 5 Best Parameters
**DT**	**criterion:** gini, entropy **max_depth:** (5, 30) **min_samples_splits:** (5, 30) **min_sample_leaf:** (5, 30) **max_features:** sqrt, log2 **splitter:** best, random	**criterion:** entropy **max_depth:** 6 **min_samples_splits:** 20 **min_sample_leaf:** 9 **max_features:** log2 **splitter:** best	**criterion:** entropy **max_depth:** 6 **min_samples_splits:** 20 **min_sample_leaf:** 9 **max_features:** log2 **splitter:** best	**criterion:** gini **max_depth:** 30 **min_samples_splits:** 26 **min_sample_leaf:** 10 **max_features:** log2 **splitter:** random	**criterion:** entropy **max_depth:** 24 **min_samples_splits:** 20 **min_sample_leaf:** 9 **max_features:** sqrt **splitter:** best	**criterion:** entropy **max_depth:** 7 **min_samples_splits:** 10 **min_sample_leaf:** 6 **max_features:** log2 **splitter:** random
**LR**	**C:** (0.01, 10.0) **solver:** newton-cg, lbfgs, liblinear, saga **penalty:** l1, l2	**C:** 0.13292918943162169 **solver:** lbfgs **penalty:** l2	**C:** 0.13292918943162169 **solver:** lbfgs **penalty:** l2	**C:** 0.13292918943162169 **solver:** lbfgs **penalty:** l2	**C:** 0.13292918943162169 **solver:** lbfgs **penalty:** l2	**C:** 0.13292918943162169 **solver:** lbfgs **penalty:** l2
**kNN**	**n_neighbors:** (1, 50) **weights:** uniform, distance **metric:** euclidean, manhattan	**n_neighbors:** 19 **weights:** uniform **metric:** euclidean	**n_neighbo:** 19 **weights:** uniform **metric:** euclidean	**n_neighbo:** 19 **weights:** uniform **metric:** euclidean	**n_neighbo:** 19 **weights:** uniform **metric:** euclidean	**n_neighbo:** 19 **weights:** uniform **metric:** euclidean
**RF**	**n_estimators:** (10,30) **max_depth:** (2, 30) **min_samples_split:** (2, 30) **min_samples_leaf:** (2, 30)	**n_estimators:** 13 **max_depth:** 6 **min_samples_split:** 3 **min_samples_leaf:** 27	**n_estimators:** 16 **max_depth:** 4 **min_samples_split:** 21 **min_samples_leaf:** 14	**n_estimators:** 17 **max_depth:** 29 **min_samples_split:** 23 **min_samples_leaf:** 19	**n_estimators:** 16 **max_depth:** 4 **min_samples_split:** 21 **min_samples_leaf:** 14	**n_estimators:** 27 **max_depth:** 8 **min_samples_split:** 7 **min_samples_leaf:** 7

**LDA**	**shrinkage:** (1e-05, 1e-04, 1e-03, 1e-2, 1e-1,0, 1)<br>**solver:** lsqr, eigen, svd<br>**tol:** (1e-05, 1e-01)	**shrinkage:** 0.001<br>**solver:** lsqr<br>**tol:** 0.0024810409748678114	**shrinkage:** 0.001<br>**solver:** lsqr<br>**tol:** 0.0024810409748678114	**shrinkage:** 0.001<br>**solver:** lsqr<br>**tol:** 0.0024810409748678114	**shrinkage:** 0.001<br>**solver:** lsqr<br>**tol:** 0.0024810409748678114	**shrinkage:** 0.001<br>**solver:** lsqr<br>**tol:** 0.0024810409748678114

Table S1-B - Hyperparameter optimization of models trained solely on acoustic voice features.

Model	Space Parameters	Fold 1 Best Parameters	Fold 2 Best Parameters	Fold 3 Best Parameters	Fold 4 Best Parameters	Fold 5 Best Parameters
**DT**	**criterion:** gini, entropy<br>**max_depth:** (5, 30)<br>**min_samples_splits:** (5, 30)<br>**min_sample_leaf:** (5, 30)<br>**max_features:** sqrt, log2<br>**splitter:** best, random	**criterion:** entropy<br>**max_depth:** 7<br>**min_samples_splits:** 10<br>**min_sample_leaf:** 6<br>**max_features:** log2<br>**splitter:** random	**criterion:** entropy<br>**max_depth:** 14<br>**min_samples_splits:** 22<br>**min_sample_leaf:** 27<br>**max_features:** log2<br>**splitter:** best	**criterion:** entropy<br>**max_depth:** 12<br>**min_samples_splits:** 12<br>**min_sample_leaf:** 18<br>**max_features:** log2<br>**splitter:** random	**criterion:** gini<br>**max_depth:** 8<br>**min_samples_splits:** 19<br>**min_sample_leaf:** 10<br>**max_features:** sqrt<br>**splitter:** random	**criterion:** entropy<br>**max_depth:** 10<br>**min_samples_splits:** 24<br>**min_sample_leaf:** 7<br>**max_features:** log2<br>**splitter:** random
**LR**	**C:** (0.01, 10.0)<br>**solver:** newton-cg, lbfgs, liblinear, saga<br>**penalty:** l1, l2	**C:** 0.013131914962817065<br>**solver:** lbfgs<br>**penalty:** l2	**C:** 0.010078244653331039<br>**solver:** newton-cg<br>**penalty:** l2	**C:** 0.012681352169084602<br>**solver:** lbfgs<br>**penalty:** l2	**C:** 0.0683210382777465<br>**solver:** saga<br>**penalty:** l2	**C:** 0.015502173243808402<br>**solver:** newton-cg<br>**penalty:** l2
**kNN**	**n_neighbors:** (1, 50)<br>**weights:** uniform, distance<br>**metric:** euclidean, manhattan	**n_neighbors:** 6<br>**weights:** distance<br>**metric:** euclidean	**n_neighbo:** 48<br>**weights:** uniform<br>**metric:** manhattan	**n_neighbo:** 10<br>**weights:** distance<br>**metric:** euclidean	**n_neighbo:** 2<br>**weights:** distance<br>**metric:** euclidean	**n_neighbo:** 10<br>**weights:** distance<br>**metric:** euclidean

**RF**	**n_estimators:** (10,30) **max_depth:** (2, 30) **min_samples_split:** (2, 30) **min_samples_leaf:** (2, 30)	**n_estimators:** 20 **max_depth:** 2 **min_samples_split:** 24 **min_samples_leaf:** 8	**n_estimators:** 30 **max_depth:** 2 **min_samples_split:** 30 **min_samples_leaf:** 21	**n_estimators:** 21 **max_depth:** 2 **min_samples_split:** 22 **min_samples_leaf:** 5	**n_estimators:** 14 **max_depth:** 3 **min_samples_split:** 4 **min_samples_leaf:** 23	**n_estimators:** 28 **max_depth:** 3 **min_samples_split:** 9 **min_samples_leaf:** 4
**LDA**	**shrinkage:** (1e-05, 1e-04, 1e-03, 1e-2, 1e-1,0, 1) **solver:** lsqr, eigen, svd **tol:** (1e-05, 1e-01)	**shrinkage:** 0.01 **solver:** eigen **tol:** 1.5339162591163613e-05	**shrinkage:** 0.01 **solver:** eigen **tol:** 1.5339162591163613e-05	**shrinkage:** 1 **solver:** eigen **tol:** 0.000534293726127977 7	**shrinkage:** 1 **solver:** eigen **tol:** 0.000534293726127977 7	**solver:** svd **tol:** 5.3370327626039544e-05

Table S1-C - Hyperparameter optimization of models trained using the difference in rankings and acoustic voice characteristics..

Model	Space Parameters	Fold 1 Best Parameters	Fold 2 Best Parameters	Fold 3 Best Parameters	Fold 4 Best Parameters	Fold 5 Best Parameters
**DT**	**criterion:** gini, entropy **max_depth:** (5, 30) **min_samples_splits:** (5, 30) **min_sample_leaf:** (5, 30) **max_features:** sqrt, log2 **splitter:** best, random	**criterion:** entropy **max_depth:** 23 **min_samples_splits:** 23 **min_sample_leaf:** 25 **max_features:** log2 **splitter:** random	**criterion:** entropy **max_depth:** 8 **min_samples_splits:** 23 **min_sample_leaf:** 28 **max_features:** sqrt **splitter:** best	**criterion:** entropy **max_depth:** 7 **min_samples_splits:** 10 **min_sample_leaf:** 6 **max_features:** log2 **splitter:** random	**criterion:** gini **max_depth:** 6 **min_samples_splits:** 17 **min_sample_leaf:** 18 **max_features:** sqrt **splitter:** best	**criterion:** gini **max_depth:** 28 **min_samples_splits:** 20 **min_sample_leaf:** 16 **max_features:** log2 **splitter:** best
**LR**	**C:** (0.01, 10.0) **solver:** newton-cg, lbfgs, liblinear, saga	**C:** 0.4323640260174416 **solver:** lbfgs **penalty:** l2	**C:** 0.0200882419015625 **solver:** saga **penalty:** l2	**C:** 0.0200882419015625 **solver:** saga **penalty:** l2	**C:** 0.010081746706252108 **solver:** saga **penalty:** l2	**C:** 0.01488262147241124 **solver:** lbfgs **penalty:** l2

	**penalty:** l1, l2					
**kNN**	**n_neighbors:** (1, 50) **weights:** uniform, distance **metric:** euclidean, manhattan	**n_neighbors:** 9 **weights:** distance **metric:** euclidean	**n_neighbor:** 42 **weights:** uniform **metric:** manhattan	**n_neighbor:** 11 **weights:** distance **metric:** euclidean	**n_neighbor:** 7 **weights:** distance **metric:** euclidean	**n_neighbor:** 16 **weights:** distance **metric:** euclidean
**RF**	**n_estimators:** (10,30) **max_depth:** (2, 30) **min_samples_split:** (2, 30) **min_samples_leaf:** (2, 30)	**n_estimators:** 19 **max_depth:** 28 **min_samples_split:** 22 **min_samples_leaf:** 27	**n_estimators:** 15 **max_depth:** 3 **min_samples_split:** 23 **min_samples_leaf:** 25	**n_estimators:** 24 **max_depth:** 8 **min_samples_split:** 27 **min_samples_leaf:** 9	**n_estimators:** 20 **max_depth:** 7 **min_samples_split:** 5 **min_samples_leaf:** 27	**n_estimators:** 16 **max_depth:** 6 **min_samples_split:** 21 **min_samples_leaf:** 14
**LDA**	**shrinkage:** (1e-05, 1e-04, 1e-03, 1e-2, 1e-1,0, 1) **solver:** lsqr, eigen, svd **tol:** (1e-05, 1e-01)	**solver:** svd **tol:** 5.3370327626039544e-05	**shrinkage:** 1 **solver:** eigen **tol:** 0.000534293726127977 7	**shrinkage:** 0.1 **solver:** lsqr **tol:** 0.03538758864779238	**shrinkage:** 0.1 **solver:** lsqr **tol:** 0.03538758864779238	**shrinkage:** 0.001 **solver:** lsqr **tol:** 0.002481040974867811 4
